# Birth Experience Mediates the Association Between Fear of Childbirth and Mother-Child-Bonding Up to 14 Months Postpartum: Findings From the Prospective Cohort Study DREAM

**DOI:** 10.3389/fpsyt.2021.776922

**Published:** 2022-01-20

**Authors:** Lara Seefeld, Victoria Weise, Marie Kopp, Susanne Knappe, Susan Garthus-Niegel

**Affiliations:** ^1^Department of Psychotherapy and Psychosomatic Medicine, Faculty of Medicine TU Dresden, Dresden, Germany; ^2^Faculty of Medicine of the Technische Universität Dresden, Institute and Policlinic of Occupational and Social Medicine, Dresden, Germany; ^3^Institute of Clinical Psychology and Psychotherapy, Technische Universität Dresden, Dresden, Germany; ^4^Faculty of Human Medicine, Institute for Systems Medicine (ISM), Medical School Hamburg, Hamburg, Germany; ^5^Department of Child Health and Development, Norwegian Institute of Public Health, Oslo, Norway

**Keywords:** fear of childbirth, pregnancy, childbirth experience, mother-child-bonding, mediation analysis, DREAM study

## Abstract

**Objective:**

To explore the longitudinal associations between prepartum fear of childbirth (FOC), birth experience, and postpartum mother-child-bonding, and the potential mediator role of the birth experience.

**Design:**

Women from the prospective cohort study DREAM completed questionnaires during pregnancy, 8 weeks, and 14 months after the birth.

**Participants:**

A community sample of *n* = 645 pregnant women from a large city in Eastern Germany participated in the study.

**Results:**

In a regression analysis, FOC predicted negative birth experience (β = 0.208, *p* < 0.001) which in turn predicted poorer mother-child-bonding both at 8 weeks (β = 0.312, *p* < 0.001) and 14 months postpartum (β = 0.200, *p* < 0.001). FOC also predicted mother-child-bonding at 14 months postpartum (β = 0.098, *p* < 0.05). Of note, this association was mediated by birth experience both at 8 weeks, indirect effect ab = 0.065, 95% CI [0.036, 0.098], and 14 months postpartum, indirect effect ab = 0.043, 95% CI [0.023, 0.067]. These effects remained stable even when adjusting for potential confounders.

**Key Conclusions:**

This study suggests that the association between FOC and mother-child-bonding is mediated by birth experience, pointing to the importance of a woman's positive subjective experience.

**Implications for Practice:**

Findings reveal two targets for peripartum interventions for women at risk for poor mother-child-bonding, namely the implementation of FOC screenings during pregnancy, and birth experience as mediating factor between FOC and mother-child-bonding. Focusing on the mother's subjective birth experience could aid to identify women at risk for impaired bonding who might need additional support.

## Introduction

With an estimated pooled prevalence rate of 14% (1), severe fear of childbirth (FOC), also referred to as tokophobia, is a common phenomenon among pregnant women, with most of the research focusing on populations from Scandinavia, Australia, and the UK (2–5). Prevalence rates for FOC vary significantly between countries and seem to have increased during the last years (1, 6). This development is especially problematic as prepartum FOC is associated with various negative outcomes for mother and child, one of them being a mother's negative postpartum rating of the birth experience (3, 7–11). Negative birth experiences have an estimated prevalence rate of 7–34% (12) and may lead to a decrease in women's self-esteem and self-efficacy, a feeling of disempowerment, and mental health problems (13). Hodnett (14) concluded in her systematic review that positive expectations seem to lead to a more positive evaluation of the birth (15), whereas negative expectations may lead to a negative evaluation (16, 17). A possible explanation for FOC predicting a more negative birth experience points to the role of endocrine stress parameters during pregnancy for the course of labor. Findings suggest that cortisol awakening response and higher plasma levels of adrenalin (which could both be influenced by FOC) interfere with uterine contractions during labor (18) and thus in turn predict a more negative birth experience (19). This explanation could be applying especially to primary FOC which describes a woman's fear before her first childbirth (20). However, endocrine stress parameters can also be influenced by the birth environment: less optimal, but modifiable circumstances (e.g., the sterile surroundings of a hospital, the perceived stress of over-worked staff, and the consequences on the communication with them) may increase women's biological stress response even if they are not affected by FOC (21, 22). By slowing down labor and increasing fetal distress, this biological stress response can further increase the possibility of medical interventions (23), such as instrumental vaginal birth or emergency cesarean section, which are also a risk factor for a negative birth experience (24, 25). Multiparous women who experienced one of those procedures as traumatic at their last birth may therefore fear the recurrence of these events during their next birth, which is referred to as secondary FOC (26–29).

Prepartum FOC seems to not only predict the level of fear during birth, but is also associated with higher levels of postpartum FOC (30–32). Accordingly, several studies have proposed the idea of a vicious cycle (33): during birth, women experience what they were already afraid of, which in turn predicts their postpartum fear and first interaction with their new-born. In support of this, Pazzagli et al. (34) found a moderate linear association between FOC and postpartum parenting stress. Further, the results of a qualitative study interviewing Swedish midwives suggest that FOC predicts both difficulties with breastfeeding and poorer mother-child-bonding (35). Although mother-child-bonding disorders have been identified as a risk factor for impaired emotional, behavioral, and cognitive development of the child (36, 37), to the best of our knowledge, there is only one quantitative study examining the association between prepartum FOC and postpartum bonding. The findings suggest a negative association between FOC and mother-child-bonding 6 weeks, but not 6 months postpartum (38) which prompts the additional question of the longitudinal development of this association.

Another factor influencing maternal bonding may be birth experience as it can have short- and long-term effects on the mother's postpartum well-being (39, 40) which in turn plays a crucial role for early bonding experiences (41). A systematic review by Bell et al. (42) also shows an association between a negative birth experience and poorer maternal postpartum caregiving. So far, the number of studies on the implications of birth experience for mother-child-bonding is small, and studies are limited to the link between symptoms of birth-related posttraumatic stress disorder (PTSD) and mostly poorer mother-child-bonding (43–47). Due to the high correlation between a subjective negative birth experience and birth-related PTSD (43), it seems likely that a negative birth experience could also predict poorer mother-child-bonding.

Other studies have focused on the mother's recalled labor pain (48) or distress during birth (45) and found significant associations with maternal bonding. As an explanation Kennell and Klaus (49) hypothesized that after a negative birth experience, mothers may be preoccupied with their own physical and emotional needs and engage less with their babies, thereby weakening mother-child-bonding.

However, the association between a women's subjective rating of her birth experience and postpartum mother-child-bonding remains understudied. If these two are indeed inter-related, enabling a positive birth experience could be a successful way to ensure a stronger bond between mother and child, thereby increasing the chance of positive child outcomes (50, 51).

At this point, a potential link between prepartum FOC, birth experience, and postpartum mother-child-bonding needs further clarification. Especially the role of a negative birth experience could be of major importance as it might emphasize prior vulnerabilities of the mother, like FOC, and increase the risk of impaired mother-child-relationships (52), therefore acting as a possible mediator between the two variables.

This study aims to explore the longitudinal associations between prepartum FOC, birth experience, and mother-child-bonding 8 weeks and 14 months postpartum. Furthermore, it will be analyzed whether the association between prepartum FOC and postpartum mother-child-bonding is mediated by birth experience.

## Materials and Methods

### Design

This study is based on data from the prospective cohort study Dresden Study on Parenting, Work, and Mental Health (“**DR**esdner Studie zu **E**lternschaft, **A**rbeit und **M**entaler Gesundheit”; **DREAM**). The DREAM study examines parental work participation, role distribution, stress factors, and how these affect peripartum outcomes and the long-term mental and somatic health of the family. Recruitment started in 2017 and finished at the end of 2020. Currently the study consists of six measurement points: T1 during pregnancy, T2 8 weeks after the anticipated birth date, T3 14 months, T4 2 years, T5 3 years, and T6 4.5 years after birth. Participants comprise a community sample of *N* = 3,865 parents from Dresden, Germany and surroundings who are expecting a child and are mainly recruited at information events of obstetrical clinics. Detailed information on the design of the study can be found in the study protocol (53).

### Sample

**Figure 1:**
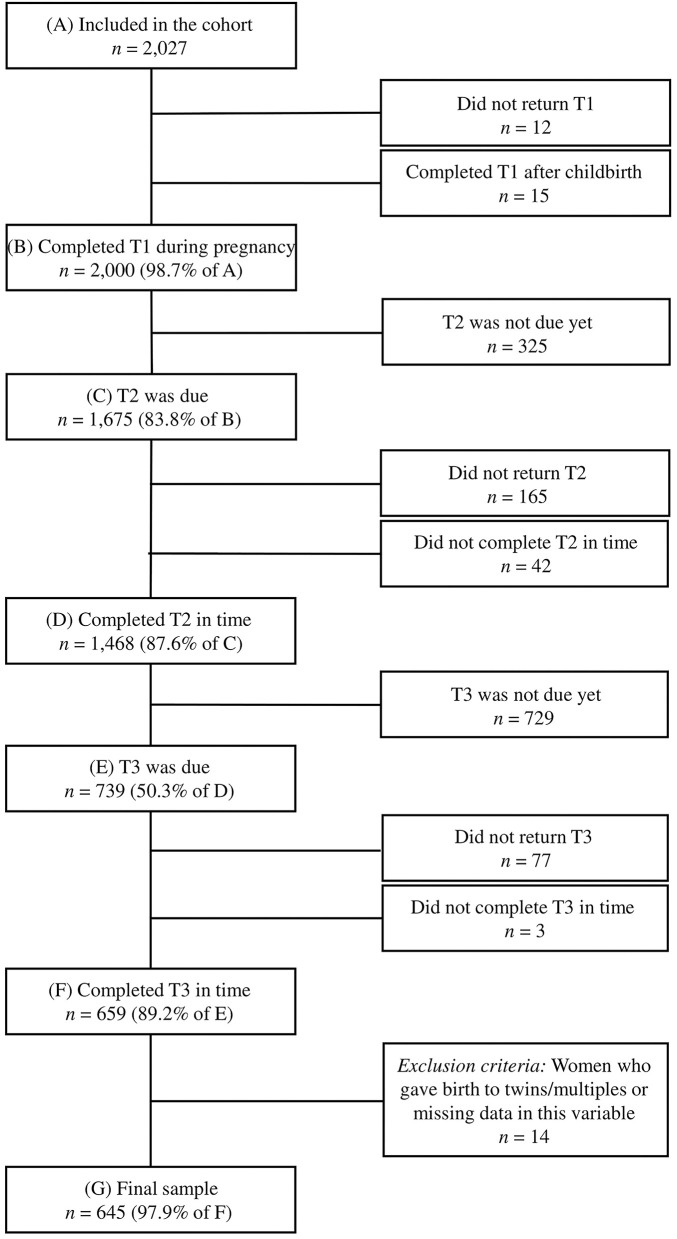
T1, during pregnancy; T2, 8 weeks after the anticipated birth date; T3, 14 months after the birth.

The present study is based on version 5 of the quality-assured data files and included data of women who gave birth to one child and completed T1, T2, and T3. At time of data extraction (17th March 2020), *n* = 2,027 women were included in the cohort. The study's retention is presented in a flow chart in Figure 1. Data from T1 were excluded when the questionnaire was completed after childbirth to ensure that prepartum FOC was measured. Additionally, data of participants who did not complete T2 or T3 within a given timeframe were excluded, because previous research has shown that the rating of the birth experience and mother-child-bonding may also depend on the time point of the questionnaire (54, 55). Therefore, data from T2 were excluded if completed earlier than 6 weeks or later than 16 weeks postpartum, and data from T3 were excluded if completed earlier than 12 months or later than 16 months after childbirth. The final sample consisted of *n* = 645 women.

### Measures

FOC was assessed using the German version of the Fear of Birth Scale [FOBS; (56)] during pregnancy (T1). The FOBS is a validated, shorter alternative (57, 58) to the widely used Wijma Delivery Expectancy Questionnaire [WDEQ-A; (59)]. The original version consists of a two-item visual analog scale in which expectant mothers are asked about their feelings concerning the approaching birth. The two items are anchored by the terms *calm/worried* and *no fear/strong fear*. In the DREAM study, each item generates scores of 0–100 with possible values being increments of 10. The two scores are then averaged to form a total score, where higher values indicate more fear. The reliability of the FOBS in the current study was excellent (Cronbach's α = 0.90).

Birth experience was assessed at T2 (8 weeks after the expected birth date) using the German version of the Salmon's Item List [SIL; (60)], a validated 20-item questionnaire that encompasses the four dimensions fulfillment, physical discomfort, emotional distress, and negative emotional experience. The items of the SIL are presented as positive and negative anchors and women are asked to rate each item on a scale from 0 to 6 depending on their feelings during the birth. The sum of all items generates the total score ranging between 0 and 120. In the original version of the SIL, higher scores indicate a more positive birth experience, but in the current analyses the items were reversed in terms of a better understanding so that higher scores indicate a more negative birth experience. The reliability of the SIL was excellent (Cronbach's α = 0.92).

Mother-child-bonding was assessed using the German version of the frequently used Postpartum Bonding Questionnaire [PBQ; (61, 62)] which screens for bonding disorders and contains 25 items on the four subscales “impaired bonding,” “rejection and anger,” “anxiety about care,” and “risk of abuse.” The PBQ asks parents (here: mothers) to think of the most difficult time with their child and to state how often they experienced each situation, with six possible answers ranging from *never* (0) to *always* (5). Item scores are added to form the scores for each subscale and a total score ranging from 0 to 125. Higher values indicate more bonding difficulties. For this study, we used data from T2 and T3. The reliability of the PBQ was excellent for both T2 and T3 (Cronbach's α = 0.90).

The following eight variables were selected as potential confounders, because they have been associated with FOC, birth experience, and mother-child-bonding in previous research: maternal age, parity, education, financial hardship, partnership satisfaction during pregnancy, maternal depressive symptoms during pregnancy, birth complications, and infant health status after birth. Except for financial hardship, birth complications, and infant health status after birth, which were measured at T2, all potential confounders were measured at T1 during pregnancy.

Education was measured by an item, which asks about the professional qualification. Participants were then divided into one group without a university degree and one group with a university degree (bachelor's degree or higher).

Financial hardship was measured by an item, which asks about financial problems during pregnancy or after the birth. It is part of a questionnaire asking about former and current critical life events and their burden based on the Life Event Questionnaire from the Avon Longitudinal Study of Parents and Children (63).

Partnership satisfaction was measured by the validated German short version of the Partnership Questionnaire (64) which comprises three subscales with three items each and an additional item assessing the general happiness of the partnership. The four possible responses range from *never/very rare* (0) to *very often* (3). The total score is generated by summation of all items and ranges from 0 to 27. The reliability of the PFB-K in the current sample was good (Cronbach's α = 0.80).

Maternal depressive symptoms were measured by the German version of the Edinburgh Postnatal Depression Scale [EPDS; (65, 66)] which assesses symptoms of depression during the past week. It consists of ten items with four possible responses respectively that are scored on a scale from 0 to 3. The total score is generated by summation of all items and ranges from 0 to 30. The reliability of the EPDS in the current sample was good (Cronbach's α = 0.83).

Birth complications and health status of the infant after birth were measured by questions based on the maternity records and the child's medical records. Complications during birth included the number of severe complications concerning the mother, e.g., failure to progress in labor, hemorrhage, perineal tear, vaginal/labial/clitoris tear, or premature or difficult abruption of the placenta. The infant's health status was measured dichotomously as complications during the first 3 days after birth (e.g., icterus, infection, hypoglycemia) which led to a hospitalization of the child.

### Data Analysis

All analyses were conducted using IBM SPSS Statistics (Version 27.0). First, data were adjusted and checked for outliers. When items from psychometric scales were missing, they were replaced by the woman's mean value in cases where <20% of the items were missing. Second, descriptive analyses (N, rates in %, mean, SD) for the sociodemographic characteristics of the sample and FOC, birth experience, mother-child-bonding, and the potential confounders were computed. Additionally, correlations between all variables were examined to identify statistically significant confounders. Third, associations between FOC, birth experience, and mother-child-bonding and the potential mediator role of birth experience were analyzed via ordinary least squares regression within the SPSS modeling tool PROCESS (67). The tool computes standardized path coefficients and the standardized total, direct, and indirect effect in a mediation model. For the confidence intervals and inferential statistics bootstrapping with 5,000 iterations and heteroscedasticity consistent standard errors (68) were used. The level of significance was set to *p* < 0.05 with 95% confidence intervals (CI). Due to missing data, *n* varied slightly between analyses.

For the interpretation of the mediated effect we followed the recommendations of Zhao et al. (69) and Rucker et al. (70) who suggest to only consider the indirect effect ab to detect mediation. According to the authors, a significant total effect between the predictor and the outcome is not a requirement for mediation, thus it was reported but not interpreted.

### Ethical Statement

All parts of the study were approved by the Ethics Committee of the Faculty of Medicine of the Technische Universität Dresden (No: EK 278062015). The couples were informed about the aims and procedures of the DREAM study, the pseudonymization of their data, and their right to withdraw from the study at any time. All participants provided written informed consent.

## Results

### Sample Characteristics

The final sample at T3 (14 months postpartum) consisted of *n* = 645 women (Table 1). The mean age of the women during pregnancy was 30.1 years (*SD* = 3.9). Most were born in Germany (96.6%), in their third trimester of pregnancy (76.6%), expecting their first child (79.3%), and living in a stable partnership (99.4%). Compared to the general population in Dresden, the women in the sample had higher education, with 60.6% holding a university degree (71). The mean scores of the FOBS, SIL, and PBQ at T2 and T3 were all below the suggested clinically relevant cut-offs, indicating low FOC, positive birth experiences, and strong mother-child-bonding, respectively.

**Table 1:** Sample description.

**Sample characteristics**	**Total (*****n*** **=** **645)**	
	***M (SD)***	**Range**
Maternal age at T1 (in years)	30.1 (3.9)	15–42
Week of pregnancy at T1	30.7 (5.8)	11–41
Age of child at T2 (in weeks)	8.5 (1.9)	6–16
Age of child at T3 (in months)	13.8 (0.5)	13–16
Fear of childbirth (FOBS score; T1)	36.6 (22.8)	0–100
Birth experience (SIL score; T2)	42.0 (21.0)	0–108
Mother-child-bonding (PBQ score; T2)	13.0 (10.2)	0–93
Mother-child-bonding (PBQ score; T3)	14.0 (9.9)	0–102
Depressive symptoms (EPDS score; T1)	5.5 (4.0)	0–23
Partnership satisfaction (PFB-K score; T1)	21.6 (4.0)	5–27
	**n^a^**	**%^b^**
**Country of birth**		
Germany	621	96.6
Other	22	3.4
**Education**		
No university degree	254	39.5
University degree	389	60.5
**Partnership status**		
Partner	637	99.4
No partner	4	0.6
**Parity**		
Nulliparous	507	79.3
Primiparous	114	17.8
Multiparous	18	2.9
**Employment status^c^**		
Full-time employed	284	44.1
Part-time employed	114	17.7
Maternity leave	93	14.4
**Number of birth complications**		
0	347	53.8
1	218	33.8
2	67	10.4
≥ 3	13	2.0
**Infant health status during the first 3 days**		
Healthy	572	89.2
Hospitalized due to complications	69	10.8
**Financial hardship**		
No financial problems	488	78.3
Financial problems during pregnancy after birth or	135	21.7

*FOBS, Fear of Birth Scale; SIL, Salmon's Item List; PBQ, Postpartum Bonding Questionnaire; EPDS, Edinburgh Postnatal Depression Scale; PFB-K, short version of the Partnership Questionnaire; T1, during pregnancy; T2, 8 weeks after the expected birth date; T3, 14 months after birth*.

a *n varies slightly due to missing data of some participants*.

b *Valid percent*.

c *Multiple answers possible*.

### Dropout Analyses

Dropout analyses were conducted for the predictor, the mediator, all potential confounders, and sociodemographic characteristics for completers vs. non-completers. Compared to completers, non-completers more often had no university degree (54.2 vs. 39.5%, χ^2^(1, *n* = 715) = 5.76, *p* = 0.016) and had a 7.40 points, 95% CI [1.51, 13.01], higher mean FOBS score than completers, *t*(708) = 2.62, *p* < 0.05, indicating more FOC. There were no differences between completers and non-completers regarding any other variable (tables on request).

### Association Between FOC, Birth Experience, and Mother-Child-Bonding

First, correlations between all variables were computed (see Table 2), revealing small to medium correlations between FOC, birth experience, mother-child-bonding, and several potential confounding variables.

**Table 2:** Correlation matrix including the predictor, mediator, outcome, and potential confounders.

	**1**.	**2**.	**3**.	**4**.	**5**.	**6**.	**7**.	**8**.	**9**.	**10**.	**11**.	**12**.
1. FOBS	–											
2. SIL	0.21^***^	–										
3. PBQ (T2)	0.13^**^	0.32^***^	–									
4. PBQ (T3)	0.14^**^	0.22^***^	0.61^***^	–								
5. Age	0.05	0.07	−0.00	−0.03	–							
6. Parity	−0.02	−0.21^***^	–0.16^***^	0.06	0.30^***^	–						
7. Education	−0.07	0.08^*^	0.13^**^	0.11^**^	0.19^***^	−0.01	–					
8. EPDS	0.41^***^	0.14^**^	0.18^***^	0.21^***^	0.02	0.09^*^	−0.08	–				
9. PFB-K	−0.04	−0.06	−0.06	–0.12^**^	−0.07	−0.27^***^	0.07	−0.20^***^	–			
10. Birth complications	−0.05	0.12^**^	0.09^*^	0.03	0.03	−0.14^**^	−0.00	−0.07	0.05	–		
11. Financial hardship	0.06	−0.03	0.04	0.10^*^	−0.10^*^	0.07	−0.16^***^	0.22^***^	−0.02	−0.05	–	
12. Infant health	0.02	0.05	0.00	−0.00	0.00	−0.08	−0.03	0.02	0.07	−0.05	0.07	–

*All associations were calculated using Pearson correlation coefficient, except for the association between dichotomous variables, which were calculated using phi coefficient. Significant correlations of potential confounders with the outcome variables, PBQ (T2) and PBQ (T3), are printed in bold. FOBS, Fear of Birth Scale; SIL, Salmon's Item List; PBQ, Postpartum Bonding Questionnaire; T2, 8 weeks after the anticipated birth date; T3, 14 months after the birth; EPDS, Edinburgh Postnatal Depression Scale; PFB-K, short version of the Partnership Questionnaire*.

* *p < 0.05*.

** *p < 0.01*.

*** *p < 0.001*.

**Figure 2:**
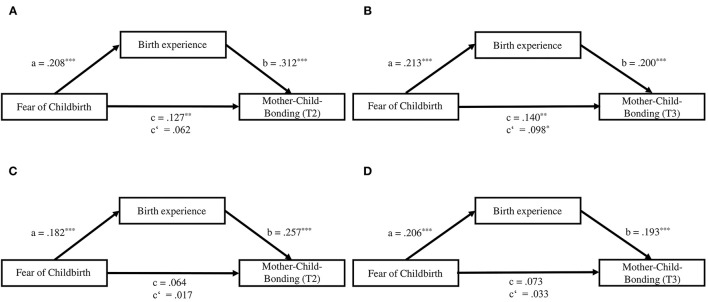
**(A)** Standardized regression coefficients for the association between fear of childbirth, birth experience, and mother-child-bonding at T2. **(B)** Standardized regression coefficients for the association between fear of childbirth, birth experience, and mother-child-bonding at T3. **(C)** Standardized regression coefficients for the association between fear of childbirth, birth experience, and mother-child-bonding at T2, controlling for maternal education, parity, prepartum depressive symptoms, and birth complications. **(D)** Standardized regression coefficients for the association between fear of childbirth, birth experience, and mother-child-bonding at T3, controlling for maternal education, prepartum depressive symptoms, financial hardship, and partnership satisfaction. c, total effect; c', direct effect. *p < 0.05. **p < 0.01. ***p < 0.001.

Second, associations between FOC, birth experience, and mother-child-bonding were examined using ordinary least squares regression within PROCESS. Figure 2A shows that higher FOC scores significantly predicted a more negative birth experience (β = 0.208, *p* < 0.001) which in turn significantly predicted poorer mother-child-bonding at T2 (β = 0.312, *p* < 0.001). While FOC had no significant direct effect on mother-child-bonding at T2, the effect was mediated by the birth experience, completely standardized indirect effect ab = 0.065, 95% CI [0.036, 0.098]. Figure 2B shows that a more negative birth experience also significantly predicted poorer mother-child-bonding at T3 (β = 0.200, *p* < 0.001). In this model, FOC had a significant direct effect on mother-child-bonding (β = 0.098, *p* < 0.05), but the effect was also mediated by the birth experience, completely standardized indirect effect ab = 0.043, 95% CI [0.023, 0.067].

Confounding variables which correlated with the two outcome variables can be found in the correlation matrix of Table 2. When maternal education, prepartum depressive symptoms, birth complications, and parity were included as confounders in the regression model with mother-child-bonding at T2 (see Figure 2C), higher FOC was still a significant predictor for a more negative birth experience, which in turn was still a significant predictor for poorer mother-child-bonding. FOC had no direct effect on mother-child-bonding at T2, but the mediated effect remained significant. The same was true for the regression model with mother-child-bonding at T3, which included the confounders maternal education, prepartum depressive symptoms, financial hardship, and partnership satisfaction (see Figure 2D). The associations between FOC and birth experience as well as birth experience and mother-child-bonding remained significant, but FOC had no significant direct effect on mother-child-bonding. Instead, the effect was significantly mediated by birth experience.

## Discussion

This study aimed to explore the longitudinal associations between prepartum FOC, birth experience, and mother-child-bonding. We found that FOC significantly predicted a more negative birth experience, which in turn significantly predicted poorer mother-child-bonding at 8 weeks and 14 months postpartum. However, FOC did not have a direct effect on mother-child-bonding. Instead, the association was mediated by birth experience.

### Association Between FOC and Birth Experience

FOC was a significant predictor for a more negative birth experience, even when adjusting for maternal education, parity, depressive symptoms during pregnancy, and birth complications (Model at 8 weeks postpartum) and maternal education, depressive symptoms during pregnancy, financial hardship, and partnership satisfaction (Model at 14 months postpartum). This finding is in line with previous research: On the one hand, endocrine stress parameters during pregnancy, like cortisol awakening response and higher plasma levels of adrenalin (which could both be influenced by FOC), may disrupt labor by interfering with uterine contractions (18) and thus in turn predict a more negative birth experience (19). On the other hand, in the current study the association between FOC and birth experience was still significant when adjusting for birth complications, suggesting that the objective birth process is only one of many explanations for the subjective postpartum birth evaluation. The way a woman's expectations of childbirth affect her perception, recall, and therefore her re-interpretation of the birth, seems to be at least equally important as the course of labor and medical interventions (32, 72). Some studies have argued that women should be encouraged to have more realistic expectations of birth to reduce negative experiences and posttraumatic stress responses (73). Instead of having a birth plan, the idea of a birth flow chart is suggested, which considers various possible events and outcomes during labor and birth (74). In contrast, other researchers highlight the importance of women's belief in birth as a natural process and their own body's capability as a way to reduce FOC and medical interventions during birth (75). Clearly, more research is needed to identify the optimal strategy for preparing women for labor and birth.

### Association Between Birth Experience and Mother-Child-Bonding

A more negative birth experience was a significant predictor for poorer mother-child-bonding, both at 8 weeks and 14 months postpartum. However, the association was stronger 8 weeks postpartum, suggesting that the impact of birth experience on mother-child-bonding weakens over time. These results are consistent with previous research (42, 45), although, to the best of our knowledge, there are no studies which examine the longitudinal development of the relationship between the subjective birth experience and mother-child-bonding. Thus, our findings emphasize the importance of a positive birth experience for the long-term bond between mother and child. Additionally, our findings support the hypothesis that not only clinically relevant birth-related PTSD symptoms may lead to poorer mother-child-bonding, but a subjectively rated negative birth experience may have the same effect. One might hypothesize that a negative birth experience influences mother-child-bonding via similar mechanisms as birth-related PTSD, because both imply negative feelings about the birth. Thus, a negative birth experience could also contribute to maternal avoidance of contact with the infant to prevent her from thinking about the negative or traumatic birth and re-experiencing it (76, 77). Furthermore, a negative birth experience may lead to a sense of failure in the mother and weaken her feeling of self-efficacy, thereby reducing her emotional availability toward the child, resulting in poorer mother-child-bonding (13, 78, 79).

### Association Between FOC and Mother-Child-Bonding and Mediator Role of Birth Experience

Except for 14 months postpartum when not considering the confounders, FOC was not a significant predictor for mother-child-bonding. At first glance, this finding contradicts previous research, which identified an association between FOC and constructs similar to postpartum mother-child-bonding. Especially the results of Klabbers et al. (38) who found a significant correlation between FOC and mother-child-bonding 6 weeks postpartum, seem to differ from our results. However, our data also showed a significant correlation 8 weeks and 14 months postpartum. It was only in the mediation analysis that it became apparent that FOC does not seem to be a direct significant predictor of mother-child-bonding, especially when considering various confounders. This is in line with further analyses by Klabbers et al. (38), in which they found no mean group differences in mother-child-bonding between women with low and high FOC, although no confounding variables were considered here. Instead, the relationship seems to be mediated by the birth experience, which, to the best of our knowledge, has not been considered in any previous studies. This indicates that FOC only influences the mother's bonding with her child via the birth experience, which is in line with the hypothesis of the vicious cycle of FOC (33): a mother who suffers from severe fear of the upcoming birth has a higher risk of experiencing a negative or traumatic birth which in turn may lead to high FOC and difficult maternal adjustment postpartum. Our study suggests that this could also affect bonding with the child even 14 months after birth.

### Strengths and Limitations

Using data from the prospective-longitudinal cohort study DREAM, we examined the longitudinal associations between FOC, birth experience, and mother-child-bonding from pregnancy to 14 months postpartum. By studying a large sample of German women, this study also contributed to the literature on FOC in Germany which is still scarce as most research focusses on populations from Scandinavia, Australia, and the UK (3, 4). Additionally, to the best of our knowledge, this was the first study that examined the association between the subjective birth experience and mother-child-bonding. Many of the previous studies have investigated the association between mother-child-bonding and symptoms of birth-related PTSD which is much less common (80, 81) than a negative birth experience [prevalence rates of 3–4% for PTSD as compared to 7–34%; (12)]. This was also the first study to examine the mediator role of birth experience for the association between FOC and mother-child-bonding, thereby further emphasizing the importance of a positive birth experience for the mother and the child. For all analyses, potential confounders were included, and only validated instruments were used to measure FOC, birth experience, and mother-child-bonding.

Nevertheless, when interpreting the results, one should keep in mind the limitations of this study. Firstly, women who dropped out of the study had higher FOC and more often had no university degree than the women in the final sample. One explanation for the higher level of FOC could be that these women were more impaired and therefore dropped out of the study, which would mean that our results underestimate the effect of FOC on birth experience and mother-child-bonding. However, completers and non-completers did not differ from each other in their birth experience, their prepartum depression scores, their partnership satisfaction, or financial hardship, making it unlikely that their impairment was the reason to drop out. Additionally, the women who participated in the study had relatively low FOC levels. Although the mean scores were similar to populations in Sweden and Australia (4, 56, 57), they were still far from the clinically relevant cut-off of 50 (56), indicating a relatively healthy sample. In general, our sample was very privileged and well educated [see study protocol, (53)], as most women were in a stable partnership, had low levels of prepartum depression, and relatively high levels of bonding with their children as the mean scores were far from the clinically relevant cut-off of 26 (82). Therefore, the current findings cannot necessarily be generalized to more impaired or clinical samples, but rather be seen as valuable insights for community samples. Finally, even though the characteristics of our sample may be an indicator for relatively low general psychiatric morbidity, we cannot exclude the possibility that apart from prepartum depression, other psychiatric comorbidities could have affected our results (83).

### Research and Practical Implications

Future research on the association between FOC, birth experience, and mother-child-bonding is needed to replicate these findings in more diverse samples including single mothers, less educated women, clinically impaired women, higher proportions of migrants, and LGBTQIA+ couples. Additionally, it should be tested whether there are differences in the described associations for nulliparous and multiparous women as parity was a significant confounder in our analyses. Regarding the improvement of women's birth experiences through altering their prepartum expectations, it should be investigated whether different strategies need to be followed for women with and without FOC. More precisely, women with FOC may profit from strengthening the belief in birth as a natural process, which their body can master, to escape the vicious circle described by Zar et al. (33). Instead, women without FOC may profit more from preparing for various unforeseen events during labor and birth and not narrowing their attention to one possible outcome specified in a birth plan. Further, the putative mechanisms by which a negative birth experience influences mother-child-bonding remain yet to be determined; some of them might be similar to those of birth-related PTSD.

Findings clearly point to the need for FOC screenings in pregnancy to identify women at risk for a negative birth experience and connected postpartum mental health difficulties. However, such screenings are not part of routine clinical practice in Germany yet (84). This might partly be due to the fact that this topic may play a subordinate role in the education of midwives and obstetricians, but also due to the immense time pressure clinicians experience during prenatal appointments, which may not leave enough room for additional questions. As a first step, FOC screenings should be included in national guidelines as a mandatory aspect of prenatal care. The FOBS is a validated instrument, which could be used for this purpose because it can be completed and interpreted quickly and is therefore suitable for the busy routines in modern practices. A further challenge, which needs to be addressed, is the effective referral of pregnant women with FOC to a specialist offering targeted intervention, like antenatal psychoeducation (85).

Additionally, women who experienced their birth as negative or traumatic need to be identified, as these mothers may need additional support in caring for and interacting with their babies as they are processing their birth experience. For this reason, it should also be investigated whether postpartum partner support can have a moderating effect on the development of bonding difficulties in mothers following a negative birth experience (86). Thus, widening the perspective to a family context rather than only focusing on the mother herself could reveal additional effective approaches for building healthier families.

## Conclusion

In this study, FOC significantly predicted a more negative birth experience suggesting that a woman's expectation of her birth might be equally important for her birth evaluation as the course of labor and medical interventions. For this reason, it could be helpful to implement FOC screenings during the routine pregnancy check-ups to refer the affected women to a specialist offering a suitable intervention. Furthermore, in this study, a negative birth experience significantly predicted poorer mother-child-bonding at 8 weeks and 14 months postpartum, although the association was stronger at 8 weeks postpartum. This stresses the importance of support for women who perceived their birth as negative and might therefore be preoccupied with emotionally processing their experience and not able to properly bond with their babies. The results of this study also suggest that the association between FOC and mother-child-bonding is mediated by the birth experience, which further emphasizes the importance of a positive birth experience for all women. It could be promising to replicate and test these findings in more diverse samples, as well as comparing them to nulliparous and parous women.

## Data Availability Statement

The dataset analyzed during the current study is not publicly available due to legal and ethical constraints, as the study's informed consent did not include public sharing of participant data. The dataset is available from the corresponding author on reasonable request. Requests to access the datasets should be directed to lara.seefeld@ukdd.de.

## Ethics Statement

This study involving human participants was reviewed and approved by the Ethics Committee of the Faculty of Medicine of the Technische Universität Dresden (No: EK 278062015). All participants provided written informed consent to participate in this study.

## Author Contributions

LS, VW, and SG-N contributed to the conception and the design of the study. VW and MK conducted data cleaning and data preparation. LS and VW performed the statistical analyses. LS wrote the first draft of the manuscript. VW, MK, SK, and SG-N wrote sections of the manuscript. All authors contributed to manuscript revision, read, and approved the submitted version.

## Funding

The DREAM study was funded by the German Research Foundation (Deutsche Forschungsgemeinschaft, DFG; Grant Numbers GA 2287/4-1 and GA 2287/4-2). This paper contributes to the EU COST Action 18211 supported by COST (European Cooperation in Science and Technology).

## Conflict of Interest

The authors declare that the research was conducted in the absence of any commercial or financial relationships that could be construed as a potential conflict of interest.

## Publisher's Note

All claims expressed in this article are solely those of the authors and do not necessarily represent those of their affiliated organizations, or those of the publisher, the editors and the reviewers. Any product that may be evaluated in this article, or claim that may be made by its manufacturer, is not guaranteed or endorsed by the publisher.
